# Radiation from UV-A to Red Light Induces ROS-Dependent Release of Neutrophil Extracellular Traps

**DOI:** 10.3390/ijms24065770

**Published:** 2023-03-17

**Authors:** Grigory Arzumanyan, Kahramon Mamatkulov, Yersultan Arynbek, Darya Zakrytnaya, Anka Jevremović, Nina Vorobjeva

**Affiliations:** 1 Frank Laboratory of Neutron Physics, Department of Raman Spectroscopy, Joint Institute for Nuclear Research, Joliot-Curie 6, 141980 Dubna, Russia; 2 Institute of Nuclear Physics, Ibragimov 1, 050032 Almaty, Kazakhstan; 3 Faculty of Physical Chemistry, University of Belgrade, Studentski trg 12-16, 11000 Belgrade, Serbia; 4 Department of Immunology, Biology Faculty, Lomonosov Moscow State University, Lenin Hills 1/12, 119234 Moscow, Russia

**Keywords:** neutrophils, photo stimulation, Raman spectroscopy, UV and visible light, cytochrome_*b*_558_, reactive oxygen species, neutrophil extracellular traps

## Abstract

Neutrophils release decondensed chromatin or extracellular traps (NETs) in response to various physiological and pharmacological stimuli. Apart from host defensive functions, NETs play an essential role in the pathogenesis of various autoimmune, inflammatory, and malignant diseases. In recent years, studies have been performed on photo-induced NET formation, mainly activated by UV radiation. Understanding the mechanisms of NET release under the influence of UV and visible light is important to control the consequences of the damaging effects of electromagnetic radiation. Raman spectroscopy was applied to record characteristic Raman frequencies of various reactive oxygen species (ROS) and low-frequency lattice vibrational modes for citrulline. NETosis was induced by irradiation with wavelength-switchable LED sources. Fluorescence microscopy was used to visualize and quantify NET release. The ability of five wavelengths of radiation, from UV-A to red light, to induce NETosis was investigated at three different energy doses. We demonstrated, for the first time, that NET formation is activated not only by UV-A but also by three spectra of visible light: blue, green, and orange, in a dose-dependent manner. Using inhibitory analysis, we established that light-induced NETosis proceeds through NADPH oxidase and PAD4. The development of new drugs designed to suppress NETosis, especially when induced by exposure to intense UV and visible light, can help to mitigate light-induced photoaging and other damaging effects of electromagnetic radiation.

## 1. Introduction

Neutrophils are the most abundant leukocytes in the circulation providing the first line of host defense against pathogens. As professional phagocytes, neutrophils contain antimicrobial enzymes in their granules and fulfill effector functions, such as phagocytosis, degranulation, and the formation of ROS in the inflammation foci. The new effector function of neutrophils, first discovered by Takei et al. [1] and extensively studied in the laboratory of A. Zychlinsky [2], is the formation of NETs, which consist of decondensed chromatin decorated with histones, granule enzymes (neutrophil elastase—NE and myeloperoxidase—MPO), and cytosolic proteins [2,3]. The process of NET formation leading to programmed cell death has been termed NETosis [4]. Subsequently, it became evident that, in addition to their protective function, NETs play an important role in the pathogenesis of inflammatory, autoimmune, and oncological diseases [5,6,7]. 

Classic or suicidal NETosis is a multistep process involving the activation, formation of ROS by the enzyme complex NADPH oxidase, and dissociation under the action of hydrogen peroxide (H_2_O_2_) of protein complexes—“azurosomes”, which are located in the membranes of azurophilic granules and contain eight different types of enzymes [8]. 

The release of serine proteases (NE, cathepsin G, and azurocidin) and MPO from azurosomes into the cytoplasm, subsequent migration of NE and MPO into the nucleus, and activation of the histone-citrullinating enzyme peptidyl-arginine deaminase 4 (PAD4) promote decondensation of nuclear chromatin [2]. These consequent stages eventually culminate in the release of chromatin outside the cell or NETosis [2].

NET formation can be induced by a large variety of physiological stimuli, such as bacteria, fungi, protozoa, viruses, and bacterial cell-wall products (lipopolysaccharides) [2]. NETosis can be activated by antibodies, cytokines (IL-8, IL-1β, and TNF-α), microcrystals, calcium and potassium ionophores, and also pharmacological stimuli, such as phorbol 12-myristate 13-acetate (PMA). In the last few years, there have been reports of NETosis stimulation by exposure to UV and the blue spectrum of visible light [9,10,11,12,13].

Every day, humans are exposed to sunlight and light that is emitted from artificial sources with different spectral characteristics and a wide range of intensities. Sunlight is a source of continuous spectrum of electromagnetic radiation that can be divided into three large groups based on wavelengths: UV radiation (200–400 nm), visible light (400–700 nm), and infrared radiation (>700 nm). 

UV is customarily divided into three wavelength groups: UV-A (320–400 nm), UV-B (280–320 nm), and UV-C (200–280 nm). The UV-C is completely absorbed by the ozone layer of the Earth’s atmosphere, while 5–10% of UV-B and 90–95% of UV-A reaches the surface of the earth [14]. The depth of penetration of UV radiation into human skin increases with its wavelength. Thus, it has been shown that UV-A reaches the lower layers of the skin (the dermis), while the shorter-wave UV-B radiation reaches the upper layer of the skin (the epidermis) and only partially reaches the dermis [15].

UV has variety of effects on the skin, one of which is the induction of photoaging [16]. Photoaging is a process in which sunlight or artificial UV radiation gradually causes clinical and histological changes in the skin—the most important of which is solar elastosis or the accumulation of elastotic material resulting from UV-stimulated degradation of collagen and elastic fibers [17]. As previously shown, neutrophils infiltrate the skin after exposure to erythemogenic doses of UV-B as well as natural sunlight [18,19,20,21,22]. To account for these data, the hypothesis was suggested [19] that neutrophils play a key role in photoaging since they are the main source of proteolytic enzymes, such as matrix metalloproteinases and neutrophil elastase, which induce the destruction of collagen and elastic skin fibers [23].

The mechanism by which proteolytic enzymes cause damage of the extracellular matrix may involve the action of ROS generated by NADPH oxidase during neutrophil oxidative burst, which inactivate blood antiproteinases (α1-proteinase inhibitor, α2-macroglobulin, and secretory leukoproteinase inhibitor) and activate the MMPs in the extracellular matrix [24,25]. In addition, it has been shown in an in vitro system that ROS formation occurs during UV radiation [9,26,27,28,29,30]. 

Thus, the analysis of the aforementioned data shows that neutrophils infiltrating the skin under the influence of UV radiation and sunlight undergo activation leading to oxidative burst (ROS formation) and degranulation (the release of MMPs and NE from azurophilic granules). As was reported in a number of studies, the sensitivity to UV radiation is a characteristic feature of patients with autoimmune diseases, such as cutaneous and systemic lupus erythematosus (CLE and SLE) [31,32], dermatomyositis, polymyositis [33,34], and sometimes Sjögren’s syndrome. 

In patients with SLE, exposure of the skin to UV radiation causes both local and systemic inflammation and is accompanied by an exacerbation of systemic disease, such as lupus nephritis [35,36]. It was shown that the photosensitization of the skin leads to systemic manifestations, which are, thus far, not well investigated. However, as was recently shown on a mouse model [22], neutrophils irradiated by UV undergo reverse transmigration from the skin to other organs, such as the kidneys, where they cause local inflammation. It is also known that neutrophils of patients with autoimmune diseases are prone to NET formation [37]. In addition, SLE patients have been shown to have increased levels of low-density granulocytes (LDG), which readily undergo NETosis [38]. Therefore, we propose that the activation of neutrophils induced by UV radiation may contribute to the enhancement of NETosis not only in patients with SLE and other autoimmune diseases but also in healthy donor patients.

To induce a photobiological effect, any radiation should be absorbed by a functional chromophore/photoacceptor molecule located in some key cell structure capable of influencing its activity and homeostasis. Redox chains are an example of such a key structure [39]. In neutrophil granulocytes, a membrane-bound heterodimeric flavohemoprotein cytochrome_*b*_558_ can be considered to be an effective photoacceptor and transducer of photo signals. Cytochrome_*b*_558_ constitutes the catalytic electron transport part of NADPH oxidase and consists of two subunits: gp91*^phox^* and p22*^phox^* [40]. The gp91*^phox^* subunit comprises binding sites for FAD (flavin adenine dinucleotide), NADPH, and two hemes required for electron transfer to oxygen. Cytochrome_*b*_558_ is the only membrane component of phagocytic NADPH oxidase producing superoxide anion radicals (O_2_^·–^) that can easily dismutate to hydrogen peroxide [41]. 

The molecule is excited electronically due to the absorption of light of a certain wavelength and, thereby, influences the primary molecular processes leading to various photobiological effects (photobioregulation). Excited states are stronger oxidizing or reducing agents compared with the ground state. The stimulation of neutrophils leads to the activation of respiratory burst, which is accompanied by an increase in oxygen consumption and the subsequent production of ROS by NADPH oxidase [42,43]. ROS are a heterogeneous group of highly chemically reactive oxygen-containing molecules, some of which are unstable and extremely reactive due to the unpaired electron. This group includes peroxides, hypochlorous acid (HClO), hydroxyl radicals (OH^+^), singlet oxygen, and superoxide anion radical.

Recently, there have been reports on the effects of electromagnetic radiation on NETosis [9,10,11,12,13]. It was shown [10] that UV-A and UV-B radiation induces sterile NETosis involving ROS generated by NADPH oxidase, which proceeds with the participation of Src, Syk, and ERK1/2 kinases as photoacceptors. In a different study [9], UV-A and blue light were reported to induce NETosis in human neutrophils that is independent on NADPH oxidase-mediated and mitochondrial ROS (mtROS). At the same time, NETosis still occurred with the participation of ROS generated in the process of extracellular excitation of riboflavin. 

In another study [11], it was reported that UV-C radiation induces NADPH oxidase-independent but mtROS-dependent suicidal NETosis and, simultaneously, the hallmarks of apoptosis, which allowed the authors to term this type of programmed cell death “ApoNETosis”.

All the aforementioned studies are inconsistent regarding the signaling pathways of NETosis induced by electromagnetic radiation. In our study, we attempt to elucidate the mechanisms of NETosis induced by electromagnetic radiation in a wide range of wavelengths, from UV-A to red visible light.

## 2. Results

### 2.1. ROS Measurements with Raman Spectroscopy

In order to confirm the involvement of ROS in NET formation in our experiments, we used Raman spectroscopy for detecting sharp peaks characteristic for hydrogen peroxide at 875–880 cm^−1^ Raman frequency (O–O stretch) and hypochlorous acid at ~732 cm^−1^. Measurements were performed in the mapping mode during the first 10–15 min after neutrophil activation by LEDs at wavelengths of 365, 405, 530, 625, and 656 nm at three doses: 4, 16, and 32 J/cm^2^. The choice of LEDs, with the exception of the 656 nm LED, was adapted to the absorption spectra bands of cytochrome_*b*_558_ [44,45].

During the mapping, the spectra were recorded in the course of a raster scan with the focused excitation beam spot across a surface area of 24 × 24 μm by using galvo-driven mirrors deflecting the beam in front of the objective. The galvanoscanner moves the focal spot across the selected sample surface area by steps of 2 μm. In each position, a Raman spectrum was accumulated by the CCD-matrix during the preset exposure time of 2 s. The spatial distribution of the power scattered into a preset spectral interval (stripe) was then calculated. This matrix is presented as a map of the signal strength.

**Figure 1:**
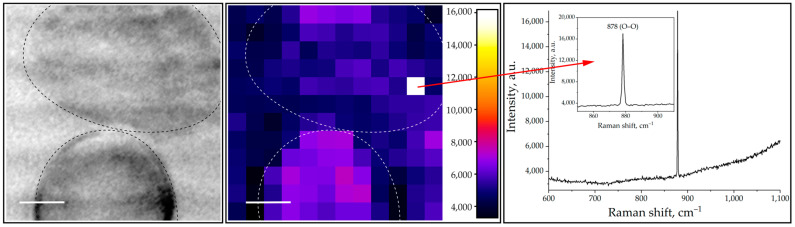
Micrograph (**left**), Raman map (**middle**), and spectrum (**right**) of neutrophils exposed to radiation with a wavelength of 625 nm and a dose of 32 J/cm^2^. The characteristic sharp lines of H_2_O_2_ can be seen on the right. The spectral gate was aligned within the interval of 872–882 cm^−1^ with excitation at 632.8 nm. Scan area 24 × 24 μm, bars: 5 μm.

In Figure 1 (on the left), one of the Raman maps obtained after the exposure of neutrophils with a 625 nm wavelength at a dose of 32 J/cm^2^ is shown. The spectral gate (stripe) was aligned within the interval of 872–882 cm^−1^ in order to capture and record the characteristic narrow peak of hydrogen peroxide. The map shows positive pixels with mostly low-to-moderate intensity at the Raman frequency of 878 cm^−1^ (H_2_O_2_ and O–O stretch) and one bright pixel with a corresponding sharp intense peak, presented in the spectrum on the right.

**Figure 2:**
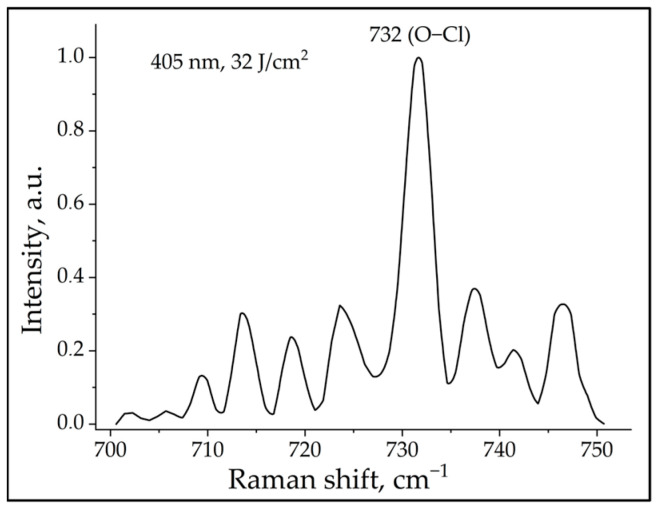
Raman spectrum of neutrophils exposed to radiation with a wavelength of 405 nm and a dose of 32 J/cm^2^. The characteristic Raman frequency of 732 cm^−1^ for HClO can be seen.

We also recorded the characteristic vibrational mode of hypochlorous acid (Figure 2). As in the case with hydrogen peroxide, the measurements were aimed to record the Raman frequency of 732 cm^–1^ (O–Cl) characteristic for hypochlorous acid. Although the signal intensity was predictably weak, it was recorded quite frequently at almost all wavelengths used in our experiment, except for the excitation of neutrophils at 656 nm wavelength.

It should be noted that, unlike O_2_**^·–^**, which has a lifetime of about 1 µs, H_2_O_2_ is more stable with a half-life of ~1 ms, and its stability depends on the pH and redox equilibrium within the cell [46]. Nevertheless, using Raman mapping, we were able to register a burst—an intense Raman peak of H_2_O_2_—as shown in Figure 1 on the right. As for hypochlorous acid, it is known to be a highly reactive compound and the main strong oxidant produced by neutrophils. We believe it is legitimate to consider both radicals, H_2_O_2_ and HClO, as spectral biomarkers of the pre-netotic state of photoactivated neutrophils. 

**Figure 3:**
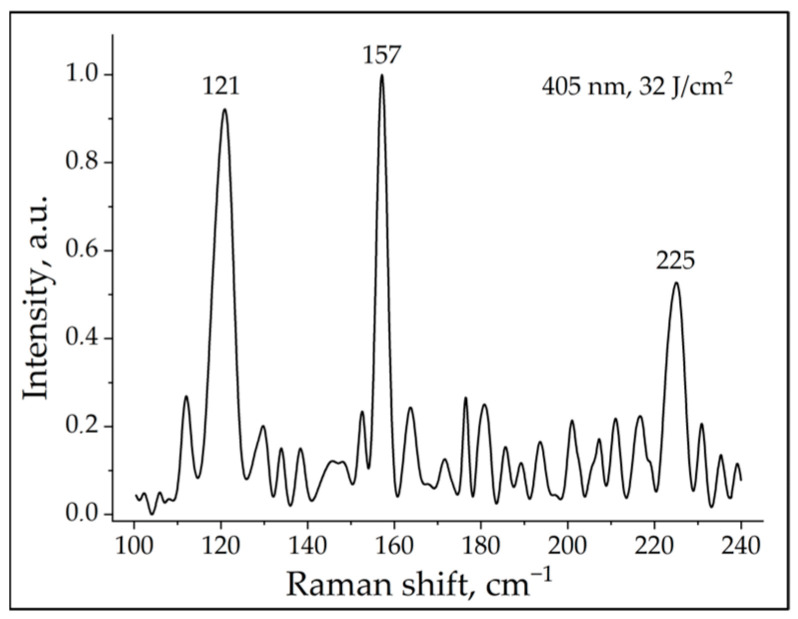
Raman spectrum of neutrophils exposed to radiation with a wavelength of 405 nm and a dose of 32 J/cm^2^. The characteristic lattice vibrational modes of citrulline can be seen.

We were also particularly interested in searching for low-frequency vibrational modes of citrulline in the Raman spectrum of photoactivated neutrophils. Low-frequency vibrational modes of the citrulline Raman spectrum were recorded upon cell photoactivation at different wavelengths except for the wavelength of 656 nm. Figure 3 shows one of such typical spectrum of citrulline with three lattice vibrational modes 121, 157, and 225 cm^−1^, when cells were irradiated at a wavelength of 405 nm. We did not observe these peaks in the negative control (unirradiated neutrophils). 

Thus, using Raman spectroscopy, we demonstrated for the first time the possibility of using this method for the registration of oxidative burst (H_2_O_2_ and HClO) and PAD4 activation. 

### 2.2. NET Formation of Photoactivated Neutrophils Detected by Fluorescence Microscopy

To investigate the ability of UV and visible light (blue, green, orange, and red) to induce NETosis, neutrophils were irradiated at room temperature in a selected dose-dependent manner: 4, 16, and 32 J/cm^2^. Stimulation with 50 nM PMA was used as a positive control, while unirradiated cells were taken as a negative control.

**Figure 4:**
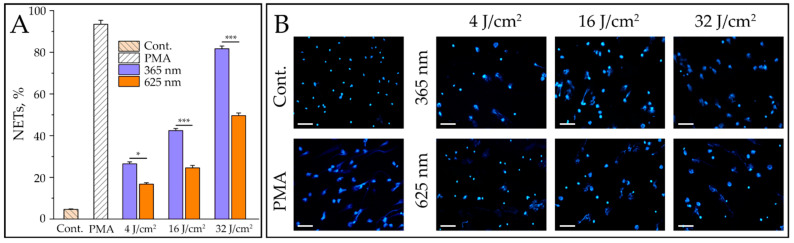
The release of NETs depending on dose at wavelengths of 365 and 625 nm. Freshly isolated neutrophils of healthy donors were irradiated with LED-lengths of 365 and 625 nm (UV-A and orange light spectrum, respectively) and doses 4, 16, and 32 J/cm^2^. NET formation was registered after 3 h incubation at 37 °C and 5% CO_2_ using fluorescence microscopy. The data represent the mean ± SD from five independent experiments (*n* = 5). Statistically significant *p* values are indicated as follows: * *p* < 0.05, and *** *p* < 0.001 (**A**). The corresponding representative images of neutrophils irradiated with LED-length of 365 and 625 nm or stimulated with PMA are shown. Bars: 50 µm (**B**).

All wavelengths, with the exception of 656 nm, tested in our study induced a dose-dependent NET release. In Figure 4A, a diagram of released NETs under neutrophil irradiation with two wavelengths (365 and 625 nm) and three doses (4, 16, and 32 J/cm^2^), as well as the results of positive (PMA) and negative controls are shown. The corresponding fluorescence microscopy images are demonstrated in Figure 4B. 

To verify NET formation under 365 nm wavelength and energy dose of 32 J/cm^2^, neutrophils were immunostained with FITC-conjugated monoclonal mouse anti-human MPO priming antibodies and DAPI (Supplementary Figure S1). The action of radiation of 365 nm wavelength (UV-A) at different energy doses on NETosis was also confirmed by biochemical measurement of extracellular DNA released into the culture medium using PicoGreen reagent (Supplementary Figure S2).

**Figure 5:**
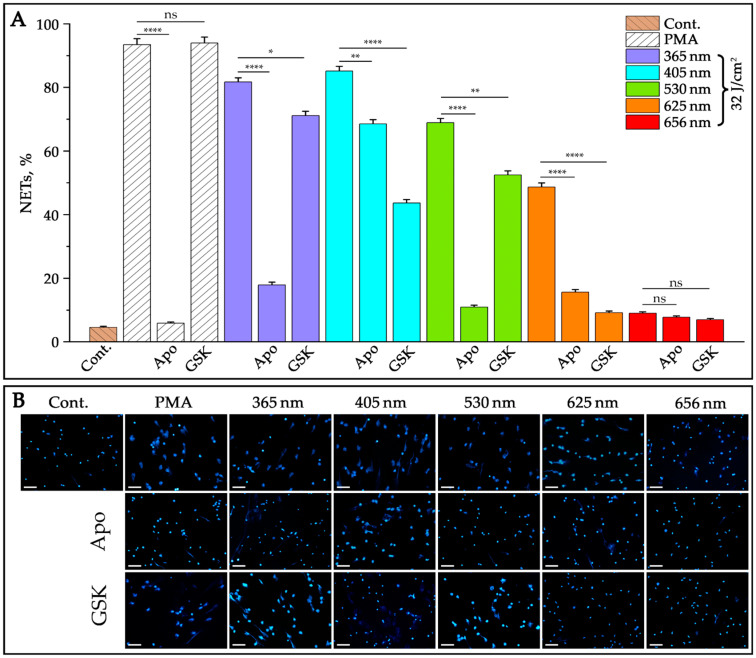
Effects of selective inhibitors of NADPH oxidase and PAD4 on NET formation in neutrophils irradiated with five LED-lengths and the same energy dose. Neutrophils isolated from the blood of healthy donors were treated with selective inhibitors of NADPH oxidase (apocynin, 400 µM) and PAD4 (GSK484, 10 µM) for 30 min. NET formation was induced by irradiation with LED-lights of 365, 405, 530, 625, and 656 nm and the same energy dose of 32 J/cm^2^. PMA (50 nM) was used as a positive control. After incubation for 3 h, the cells were stained with DAPI and analyzed using fluorescence microscopy. The data represent the mean ± SD from five independent experiments (*n* = 5). Statistically significant *p* values are indicated as follows: * *p* < 0.05, ** *p* < 0.01, and **** *p* < 0.0001. Insignificant differences are marked as “ns” (**A**). Representative fluorescence images of NET formation after neutrophil treatment with inhibitors and irradiation with LED-lengths are shown. Scale bars: 50 µm (**B**).

We also wanted to elucidate whether NADPH oxidase is involved in NET formation induced under light radiation. To test this, we used a specific inhibitor of NADPH oxidase—apocynin. As can be seen in Figure 5, apocynin inhibits NET formation induced by UV-A and three wavelengths of visible light (blue, green, and orange), indicating the involvement of NADPH oxidase in light-induced NETosis.

One of the hallmarks of NET formation is the dependence on the activity of enzyme involved in histone citrullination PAD4. Therefore, we also wanted to elucidate whether PAD4 is engaged in light-induced NET formation. For this, we used a specific inhibitor of PAD4, GSK484. As shown in Figure 5, the incubation of neutrophils with GSK prior to light irradiation caused moderate suppression of NETosis, indicating the involvement of PAD4 in light-induced NET formation. We also noticed that NET release during photostimulation was faster compared to PMA-induced NETosis. However, in this study, we did not conduct a detailed kinetic analysis of NET formation leaving this issue for the next stage of research.

## 3. Discussion

In the present study, we focused on the role of radiation with different wavelengths to induce NET formation on the model of human neutrophils. We discovered that UV-A and three spectra of visible light (blue, green, and orange) induced the release of NETs. Using specific inhibitors of NADPH oxidase and PAD4, we demonstrated that these mediators of classical NETosis are involved in NET release induced by light radiation. Therefore, ROS formation and histone citrullination contribute to chromatin decondensation in this setting. 

**Figure 6:**
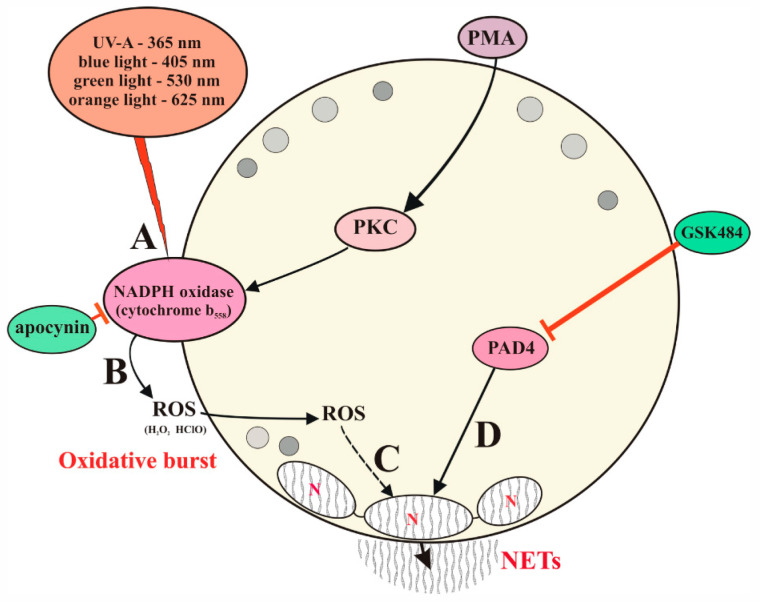
The proposed mechanism of NETosis induced by UV-A and wavelengths of visible light. (**A**) Cytochrome_*b*_558_, being the catalytic electron transport part of NADPH oxidase, absorbs UV-A, blue, green, and orange light, which leads to the activation of NADPH oxidase and the following ROS generation (**B**). ROS activate the translocation of enzymes NE and MPO from the azurosomes of azuphilic granules to the cytoplasm and their subsequent migration to the nucleus (**C**), where—together with PAD4 (**D**)—they promote the decondensation of nuclear chromatin and NETosis. Inhibition of NADPH oxidase by apocynin and PAD4 by GSK484 leads to suppression of NETosis.

The most likely photoacceptor and transducer of photo signals, in our opinion, is the membrane-bound heterodimeric flavohemoprotein cytochrome_*b*_558_, a structural component of NADPH oxidase, which contains redox centers; however, this hypothesis has not been proven directly. In our study, Raman spectroscopy was used to register oxidative stress and ROS generation for the first time. Characteristic Raman frequencies were recorded for two radicals: H_2_O_2_ (878 cm^−1^) and HClO (732 cm^−1^). In addition, low-frequency Raman spectroscopy detected three lattice vibrational modes for citrulline in the irradiated samples. Thus, Raman spectroscopy can be considered as a novel method for detecting the oxidative burst and activation of citrulline in the process of NET formation of human neutrophils. The proposed mechanism of light-induced NETosis is presented in the form of a diagram in Figure 6.

As shown in a recent study [9], UV-A and blue-light induced NETosis in human neutrophils independently of NADPH oxidase-mediated and mitochondrial ROS. At the same time, according to the authors, NETosis still occurred with the participation of ROS, which were generated in the process of extracellular excitation of riboflavin. In contrast to this study, we recorded sharp peaks of ROS and citrulline in the process of neutrophil irradiation and showed a decrease of NETosis upon NADPH oxidase inhibition, indicating the involvement of intracellular ROS during light irradiation. 

In a different study [11], UV-C was reported to induce NADPH oxidase-independent but mtROS-dependent suicidal NETosis. Simultaneously, the authors registered hallmarks of apoptosis, which allowed them to term this type of NETosis “ApoNETosis”. Unfortunately, the authors did not indicate the nature of photoacceptor for UV-C. It should be noted that UV-C is a high-energy light that can induce extremely different methods of NET formation. However, the model used by the authors is artificial since UV-C radiation is almost completely absorbed by the ozone layer of the Earth’s atmosphere. 

Another study reported [10] that UV-A and UV-B induce NADPH oxidase-dependent NETosis of human neutrophils. Interestingly, the authors stated that enzymes, such as Src-kinases, phospholipase A2, and ERK1/2, can be photoacceptors in this model. It is well known that the penetration of light through the human skin depends, to a large extent, on the wavelength. About 10–15% of UV-A and 40–50% of blue light can pass through the epidermis and reach deeper layers [47]. In human skin, the light transmission depends on reflection and absorption, while the depth of light penetration increases with increasing wavelength. However, the actual penetration of each wavelength is also highly dependent on the specific composition of the skin, body area, age, sex, skin type, pigmentation, and therefore ethnicity. Persons with delicate, slightly pigmented skin, children, as well as those suffering from Graves’ disease and vegetative dystonia are more sensitive to light.

Neubert et al. [9] observed NET formation after the irradiation of neutrophils starting from 18 J/cm^2^ at 375 nm wavelength. This dose corresponds to approximately 40 h of exposure under the sun [9]. At almost the same wavelength of radiation (365 nm, as in our case) and approximately twice a dose (32 J/cm^2^), the time spent under the sun is correspondingly reduced. It should be noted that, in reality, it is necessary to consider a much wider spectrum of sunlight that is not limited to individual wavelengths as well as factors, such as the thickness of the local ozone layer, location on the earth, weather conditions, radiation angle, height above sea level, time of the day, and season, which will affect the penetration of light [48]. 

These factors are certainly important; however, it should be considered that human skin is simultaneously exposed to a wide spectrum of sunlight with different wavelengths, and this can lead to the absorption of photons by various chromophores with different redox states. Therefore, it is reasonable to assume that cytochromes with different absorption may play various roles in the generation of subsequent photobiological mechanisms. At the same time, the intensity of UV and visible radiation of sunlight varies over the day, and this, in turn, complicates in vivo experiments. 

For many decades, UV light has been considered as an inhibitor of adaptive immunity [49]. The mechanisms governing UV-mediated immunosuppression in the skin are complex and include down regulation of the effector CD4^+^ T-lymphocytes and memory CD4^+^ T-lymphocytes [50] as well as recruitment of activated neutrophils producing IL-10 [51]. However, UV light is also a well-known trigger for skin inflammation in susceptible autoimmune patients, especially those with systemic and cutaneous lupus erythematosus [31,32], dermatomyositis [33,34], and Sjögren’s syndrome. 

Such opposite effects of UV radiation in healthy people and patients with autoimmune diseases are due to the peculiarities of immune reactions that occur in the skin of healthy and immunocompromised people. In particular, it has been shown that neutrophils isolated from the blood of SLE patients as part of the mononuclear cell fraction during density-gradient centrifugation—the so-called low-density granulocytes—produce an excess of NETs, which are associated with SLE and CLE lesions [37,38]. In addition, it was shown that skin exposure to high doses of UV-B stimulated neutrophil transmigration to the kidneys, where they promoted inflammation and kidney damage [22]. 

Thus, although the formation of NETs in the skin of healthy people exposed to electromagnetic radiation does not pose a great threat, in immunocompromised patients with systemic and cutaneous lupus erythematous and dermatomyositis, radiation can cause an aggravation of the underlying autoimmune diseases due to NET formation. 

Therefore, the development of new drugs designed to suppress NET formation, especially in areas exposed to UV and visible light, is extremely important for prevention and the recovery of patients with autoimmune diseases.

## 4. Materials and Methods

### 4.1. Reagents

PMA (PKC activator) and GSK484 (PAD4 inhibitor) were purchased from Sigma-Aldrich (Sigma-Aldrich, Inc., St. Louis, MO, USA). Apocynin (NADPH oxidase inhibitor) and DAPI (DNA dye) were obtained from Abcam (Cambridge, UK). Ficoll-Hypaque with densities of 1.119 and 1.077 g/cm^3^ and RPMI 1640 medium containing L-glutamine were purchased from PanEco Ltd. (Moscow, Russia).

### 4.2. Isolation of Primary Human Neutrophils

Peripheral blood was collected from healthy volunteers according to the recommendations of the Ethical Committee of the Biology School of Moscow State University. Fully informed consent was obtained, and all investigations were conducted according to the principles laid down in the Declaration of Helsinki. Three co-authors of the present publication (G.A., Y.A., and K.M.) were donors for this work. Neutrophils were isolated by centrifugation in a double-density gradient using Ficoll-Hypaque (1.077 and 1.119 g/cm^3^) at 400 g for 40 min at room temperature (RT) as previously reported [52]. 

After washing the neutrophils in phosphate-buffered saline (PBS), the contaminating erythrocytes were lysed in bidistilled water for 1 min followed by restoration of isotonicity with concentrated PBS. Isolated neutrophils were suspended in RPMI 1640 supplemented with 2 mM L-glutamine to the concentration 1 × 10^5^ cells/mL. The choice of medium free of photosensitive substances, such as serum, avoids light-induced unwanted cell reactions. Microscopic evaluation of the isolated cells revealed that >97% were neutrophils. Cell viability was not <98% as judged by Trypan blue exclusion. 

### 4.3. Neutrophil Irradiation

Selected neutrophils were irradiated with WheeLED wavelength-switchable LED sources (Mightex, Toronto, ON, Canada) at the following wavelengths: 365 nm (UV, WLS-LED-0365-04), 405 nm (blue light, WLS-LED-0405-03), 530 nm (green light, WLS-LED-0530-03), 625 nm (orange light, WLS-LED-0625-03), and 656 nm (red light, WLS-LED-0656-03). A frosted quartz scattering plate was used during irradiation for the uniform distribution of radiation over the surface of the samples. The cells were irradiated at RT in a selected dose-dependent manner: 4, 16, and 32 J/cm^2^. The irradiation power of the LEDs was measured using a PM100A powermeter (Thorlabs, Newton, NJ, USA).

The cell suspension was added in a volume of 100 μL to each well of a 96-well plate (Corning Inc., Somerville, MA, USA) and left to settle for 15 min for adhesion. Before, during, and after light irradiation or PMA-mediated activation, the cells were carefully shielded from other light sources. After activation, the cells were incubated for 3 h (37 °C, 5% CO_2_) in an incubator (N-Biotek NB-203, Bucheon-si, Republic of Korea) followed by fixation with 4% glutaraldehyde.

### 4.4. Raman Spectroscopy

To measure the spectra of radicals, particularly hydrogen peroxide and hypochlorous acid, as well as the spectrum of citrulline in the low wavenumber range, Raman spectroscopy was applied. Confocal microspectroscopy setup with high spectral resolution and high scanning speed of the laser spot was used for both localized spectral measurements of spontaneous Raman scattering and mapping under excitation by a 633 nm helium–neon laser. This included a scanning laser spectrometer “Confotec CARS” (SOL Instruments LLC, Minsk, Belarus) coupled with an inverted microscope NIKON TE2000-E (Nikon, Tokyo, Japan).

The samples were placed on a motorized sample positioning stage (Prior Scientific, Cambridge, UK, H117TE). The laser beam was focused on the sample with Olympus 40× lens (NA-0.6) to a spot of ~1 μm. All Raman spectra were collected in the backscattering geometry and dispersed by a 600-grooves-per-millimeter diffraction grating mounted in the MS520 monochromator–spectrograph. A Peltier-cooled charge-coupled device camera (ProScan HS-101H) was used for detection of the spectra collected at different localizations of the analytes.

### 4.5. NETs Visualization with Fluorescent Microscopy

To examine NET formation, the cells were washed twice with PBS, and then neutrophil DNA was stained with DAPI (10 µM) for 10 min. After staining, the cells were washed with PBS. Images were taken in a 96-well plate with a Nikon Eclipse Ts2R-FL fluorescent LED microscope using NIS-Elements BR software 5.30.05, an Epi-FL C-LED385 filter, and a CFI Super Plan Fluor ELWD ADM 20× objective with a 0.45 numerical aperture and a working distance of 8.2–6.9 mm. Twenty frames were captured in each well. The total number (by 20 frames) of netotic and intact cells was counted using ImageJ software 1.53q.

### 4.6. Statistical Analysis

Statistical analysis was performed using GraphPad Prism (version 9.0 for Mac or Windows, GraphPad Software Inc., San Diego, CA, USA). The statistical significance was assessed by one-way ANOVA followed by Bonferroni’s and Tukey’s multiple comparisons tests. The data are expressed as the mean ± SD. Statistically significant *p* values are indicated in the figures as follows: * *p* < 0.05, ** *p* < 0.01, *** *p* < 0.001, and **** *p* < 0.0001. 

## Data Availability

The data that support the findings of this study are available on re-quest from the authors.

## Supplementary Materials

The following supporting information can be downloaded at: https://www.mdpi.com/article/10.3390/ijms24065770/s1.
