# Supplementary material for: Radiation from UV-A to Red Light Induces ROS-Dependent Release of Neutrophil Extracellular Traps
Source: Int J Mol Sci. 2023 Mar 17;24(6):5770. doi: 10.3390/ijms24065770 (PMC10051944; doi:10.3390/ijms24065770)
Supplement: Supplementary file 1 [file ijms-24-05770-s001.zip › ijms-2258383-supplementary.pdf]

Supplementary material

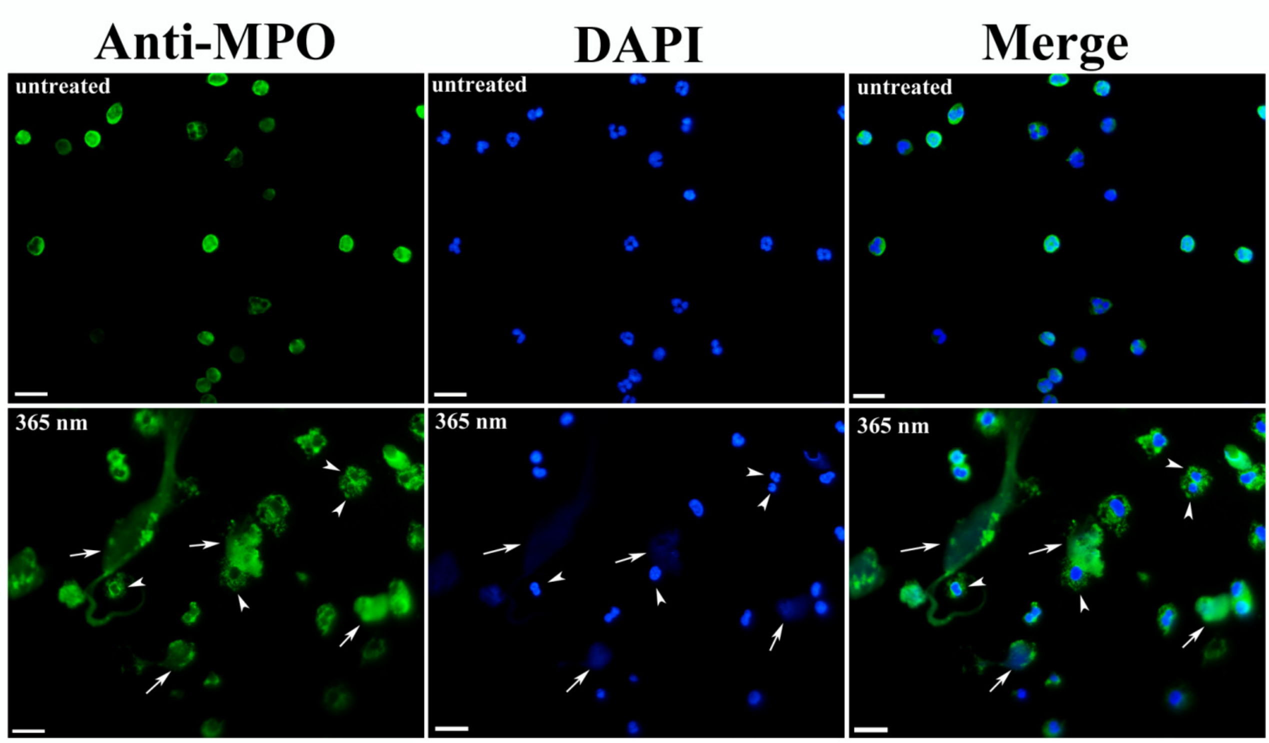

Supplementary Figure S1. Immunofluorescence images of intact and irradiated human neutrophils. Irradiation of human neutrophils at UV-A (365 nm wavelength) with energy dose of 32 J/cm^2 caused NETs formation. For immunofluorescence staining, neutrophils were fixed with 4% glutaraldehyde for 10 min, washed with PBS, and permeabilized using 0.1 % Triton X-100 in PBS for 2 min at room temperature. Nonspecific binding was reduced by pre-incubation of cells with blocking buffer (including human immunoglobulins) for 20 min. Immunofluorescence staining was performed with FITC-conjugated monoclonal mouse anti-human MPO priming antibodies (Invitrogen, USA). Blue color: staining of chromatin with DAPI. Green color: staining of MPO with FITC-conjugated anti-MPO monoclonal antibodies. NETs-forming cells are indicated with arrows, intact cells are indicated with arrow heads. Scale bars, 25 μm.

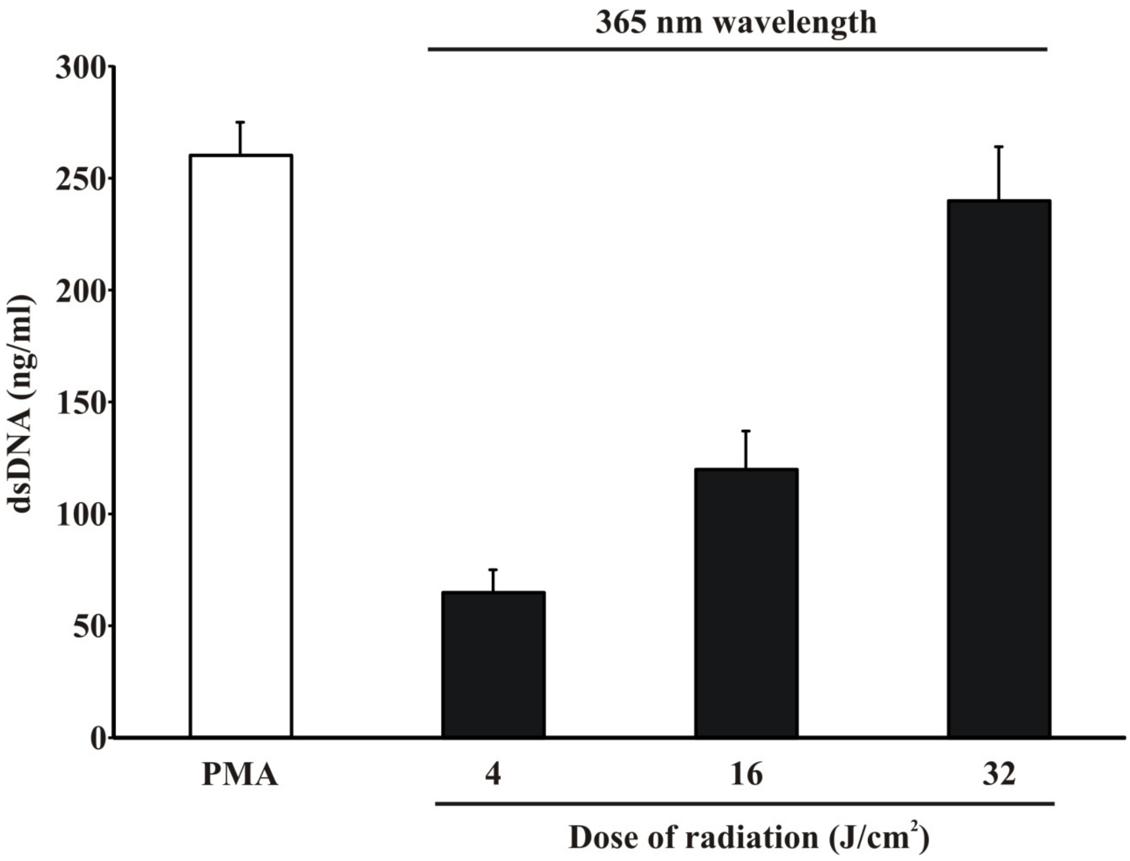

Supplementary Figure S2. Quantification of released dsDNA in culture supernatants determined by PicoGreen reagent.

Human neutrophils were seeded on the 96-well plate at 1 x 10^5 cells per well (100 µl). NETs formation was induced by irradiation of neutrophils at 365 nm wavelength (UV-A) with different energy doses. After irradiation, 1.5 U of micrococcal nuclease (Worthington) was added to the wells and plate was incubated for another 15 min at 37°C. The culture supernatant of each well (100 µl) was collected, adjusted to 2.5 mM EDTA, centrifuged at 300 g for 5 min to remove cellular debris, and saved for analysis of DNA. DNA content of each supernatant was measured with Quant-iT™ PicoGreen dsDNA Assay Kit (Molecular Probes, Invitrogen) using ELx808 Absorbance Microplate Reader (Bio-Tec Instruments Inc., USA). The excitation wavelength and emission wavelength were 485 nm and 535 nm, respectively. The DNA concentration was calculated using a standard curve generated from a series of Lambda DNA standard provided by the manufacturer. Data represent mean ± SD from five independent experiments (n=5).
